# Postural orthostatic tachycardia syndrome and post-acute COVID-19

**DOI:** 10.21542/gcsp.2022.13

**Published:** 2022-06-30

**Authors:** Youssra Amekran, Narjisse Damoun, Abdelkader Jalil El Hangouche

**Affiliations:** Department of Physiology, Faculty of Medicine and Pharmacy of Tangier, Abdelmalek Essaadi University, Tangier, Morocco

## Abstract

While the acute illness of COVID-19 was the initial focus of concern, there are increasing reports of patients with chronic symptoms, known as long-COVID. Dysautonomia may be a possible post-acute neurological complication explaining the persistent symptoms observed in long COVID. Postural tachycardia syndrome (POTS), a form of dysautonomia characterized by sustained tachycardia and orthostatic intolerance, has been increasingly reported in patients after SARS-CoV-2 infection. In this context, this review aimed to report and discuss the available literature pertaining to post COVID-19 POTS.

## Introduction

Coronavirus disease 2019 (COVID-19), caused by severe acute respiratory syndrome coronavirus 2 (SARS-CoV-2), has spread worldwide since December 2019. The COVID-19 pandemic is described as a multi-organ disease, and the acute phase of infection results in a broad spectrum of manifestations ranging from asymptomatic and mild symptoms to severe outcomes and death^1–4^.

While medical attention has been focused on the management of acute illness, an increasing number of patients have experienced symptoms such as fatigue, dyspnea, chest pain, palpitations, cognitive disturbance and orthostatic intolerance, long after their acute infectious period^3,5^. These persistent symptoms have been termed post-acute COVID-19 syndrome or long-COVID^6^. Among these symptoms, several are associated with neurological disorders, notably postural orthostatic tachycardia syndrome (POTS)^6^.

POTS is a blood-circulation disorder that affects the autonomic nervous system^7^. Its principal feature is orthostatic intolerance and is characterized by a sustained heart rate increment of 30 beats/min or more in the absence of orthostatic hypotension (OH)^7–9^.

The most commonly associated symptoms include fatigue, nausea, dizziness, light-headedness, palpitations, chest pain and exercise intolerance^8,10–14^. Data on the worldwide prevalence of POTS are missing; however, an estimation of 0.1 to 1% is the most frequently reported in the literature based on estimations in the United States^12,13^.

Females are affected more often than males with F:M ratio > 4:1^12,13^.

POTS has largely been involved in post-viral symptomatology^15–18^, with 28–41% of POTS cases reported to be associated with viral infections^19,20^, suggesting post viral autoimmune activation is a possible pathophysiological mechanism^16^. With an increasing number of patients being described with a wide variety of symptoms, particularly those implicated in autonomic dysfunction, long after their acute COVID-19 infectious period, we aimed to review the available literature on post COVID-19 POTS cases.

## Methods

A literature search was conducted to identify relevant literature pertaining to post COVID-19 POTS, using PubMed, Scopus, Web of Science and Embase databases. The keywords used were: “Postural Orthostatic Tachycardia Syndrome”, “POTS”, “dysautonomia”, “autonomic dysfunction”, “novel coronavirus”, “severe acute respiratory syndrome coronavirus 2”, “SARS-CoV-2”, ‘’Corona virus disease pandemic”, “COVID-19”, “2019-nCoV”. We included articles published in English from the period between January 2020 and November 2021. A total of 9 papers were found.

## Results

### Post COVID-19 postural orthostatic tachycardia syndrome

POTS is one of the most common autonomic disorders and is characterized by a wide range of clinical manifestations, including lightheadedness, palpitations, blurred vision, headaches, dyspnea, exercise intolerance, fatigue, gastrointestinal symptoms, pain, near-syncope, and syncope, resulting in a reduced daily functional capacity^21^.

POTS is defined as an increase in HR of ≥30 bpm when moving from a recumbent to an upright posture within 10 min of standing (or ≥40 bpm in individuals of 12–19 years of age), without significant OH^22–24^. Acute stressors have been reported to trigger POTS, including immunological triggers such as viral infection, frequent upper respiratory or gastrointestinal tract infections, vaccination, pregnancy, surgery, and traumatic events^15,19,25^. Viral infection is the most commonly reported and has been shown to trigger POTS in 28–41% of patients^19^. Various infectious pathogens have been suggested to be associated with the onset of POTS, including the Epstein Barr virus^25–27^, upper respiratory infections, gastroenteritis and recently SARS-CoV2^17,28,29^.

After reviewing the literature on patients with persistent symptoms following SARS-COV-2 infection and diagnosed with POTS, a summary of patient characteristics is shown in Table 1. A total number of 26 patients were included. The age of the patients ranged from 22 to 59 years, and the majority of cases were females (69%), with prominent symptoms that emerged several weeks after acute infection and lasted beyond months. The most common symptoms included fatigue, palpitations, chest pain, orthostatic intolerance, exercise intolerance and cognitive impairment (brain fog). Gastrointestinal complications and mast cell activation symptoms were observed at a lower frequency. Autonomic function was evaluated using the active stand test and head-up tilt table (HUT). Valsalva test (with HUT) and quantitative sudomotor axon reflex tests (QSART) were also performed.

**Table 1:** Summary of published cases of post COVID-19 POTS.

Reference	Patients	Symptoms	POTS diagnosis time following the acute COVID-19	Tests and diagnosis
Johansson M. et al. [66]	Patient 1: 42-year-old woman	Unable to stand more than5 min, palpitations, dizziness, heat, and exercise intolerance.	5–6 months	HUT showed a HRincrement of 50 bpm with initial OH, and Valsalva maneuver showed a hyperadrenergic response.
	Patient 2: 28-year-old woman	Chest pain, fatigue, nausea, headache. gastrointestinal symptoms, and orbital edema.	3 months	Active standing testshowed a HR increase of 53 bpm without BP fall, and HUT revealed symptomatic sinus tachycardia superior to 130 bpm without BP fall.
	Patient 3: 37-year-old woman	Fatigue, muscle weakness, insomnia, palpitations, brain fog with trouble concentrating.	Not specified	Active standing testshowed a HR increment of 44 bpm without BP fall.
Kanjwal M et al. [28]	Patient: 36-year-old woman	Fatigue, headache, dizziness, chest pain, and palpitations, particularly while getting up from sitting position.	3-4 weeks	HUT showed a HRincrement of 41 bpm without any fall.
Miglis MG et al. [29]	Patient: 26-year-old woman	Tachycardia, chest pain, shortness of breath, fatigue, exercise intolerance, diarrhea, tremors, and worsening restlessness.	3 months	HUT demonstrated aHR increase of 65 bpm with episodic hypertensive systolic BP surges to 170 mmHg in an oscillatory pattern, and robust BP responses to Valsalvamaneuver pattern in the absence of hyperventilation, indicating the presence of a n hyperadrenergic state.
Blitshteyn S al. [67]	Patients: 15 patients, mean age: 42.4 years old (min: 25; max: 59) with predominance of females 6M/9F.	Tachycardia, fatigue, hypersomnolence, anosmia, ageusia, exercise intolerance, chest pain, headache, fever, dizziness, weight loss, diarrhea and presyncope.	6–8 months after COVID-19	10-min stand test or TTTs.
Umapathi T et al. [68]	Patient: 39-year-old man	Tachycardia, dry mouth, pain and vibration sense.	18 days	Active standing testdemonstrated a HR increase of 35 bpm and an increase of 31 bpm after passive60-degree tilt (5min) without decrease in blood pressure.
Shouman K et al. [69]	Patient 1: A 35-year-old woman	Constant tingling in the feet, orthostatic lightheadedness, brain fog, orthostatic intolerance and presyncope	2 months	Tilt test showed a HR increase of 31 bpm.
	Patient 2: A 35-year-old woman	Orthostatic lightheadedness, tachycardia, and hyperhidrosis	4 months	Autonomic reflex screen revealed excessive heart rateacceleration during tilt meeting POTS criteria, with hyperadrenergiccharacteristics including an increase in bloodpressure on tilt and resting sweat activity on QSART.
Ishibashi Y et al. [70]	Patient: A 25-year-old woman	Fatigue, palpitations,dyspnea and chest pain, especially while getting up from sitting position.	3 weeks	Tilt test showed an increase in heart rate from 80 bpm in the supine position to 110 bpm in the absence of hypotension during a 10-min head-up tilt at an angle of 90°, which returned to the baseline level immediately after lowering to the supine position.
Schofield JR et al. [71]	Patient: A 50-year-old woman	Nausea, presyncope, dyspnea, chest tightness, myalgia, constant thirst, pruritus, and temperature dysregulation.	Approximately 2 months	Active standing test showed an increase in HR from supine to standing of 55 bpm.
O’Sullivan JS et al. [10]	Patient: A 22-year-old woman	Dyspnea and chest tightness.	3 weeks	Active standing test revealed a HR increase of 30 bpm.

BP: blood pressure; HR: Heart rate; HUT: head-up tilt; OH: Orthostatic hypotension; QSART:quantitative sudomotor axon reflex test; TTT: Tilt test table

### POTS management

The management of POTS is challenging as there are no uniformly recommended therapies, and randomized clinical trials for targeted treatments are scarce^21,24^. The currently available management of POTS (besides accurate diagnosis) includes non-pharmacologic and pharmacologic approaches, in combination with patient education about alleviating the symptoms, long-term prognosis, and available therapeutic options^21,30^.

Regular, structured, progressive, and supervised exercises have been described as effective non-pharmacological treatments to improve POTS-related symptoms^9,21,30,31^. However, given the possible activity-induced symptoms, exercise should be restricted to no upright exercises such as swimming, rowing machines, and recumbent cycles^9,21,24,30,31^. A previous progressive 3-month exercise regimen was effective in reducing the standing heart rate and alleviating symptoms^32^.

Other non-pharmacological interventions include increased fluid and salt intake, physical counter maneuvers (muscle contraction, leg crossing)^33^, compression devices, avoidance of medication worsening POTS, and avoidance of POTS symptom exacerbation such as caffeine and alcohol intake and heat exposure^21,24,34^.

Pharmacological treatments for POTS are considered first-line POTS management^35^. In addition, they are not proven to be more effective than non-pharmacological treatments and should be used with caution, given their probable side effects^30^. The most widely used medications include fludrocortisone, which is a synthetic mineralocorticoid aldosterone analog utilized to increase salt retention and plasma volume,^9,30^, alpha-1-adrenergic agonist midodrine resulting in systemic vasoconstriction and increasing venous return and effective in hypotensive phenotypes, clonidine and alpha-methyldopa, beta-blockers (propranolol, metoprolol), and pyridostigmine^24,30^.

Some drugs may worsen some specific symptoms such as tachycardia, including selective serotonin and/or norepinephrine reuptake inhibitors, amphetamines and droxidopa or exacerbate orthostatic intolerance including calcium channel blockers, diuretics, nitrates, and opiates. However, they may be effective in some patients in accordance with their history and clinical presentation^30,36^.

**Figure 1.**
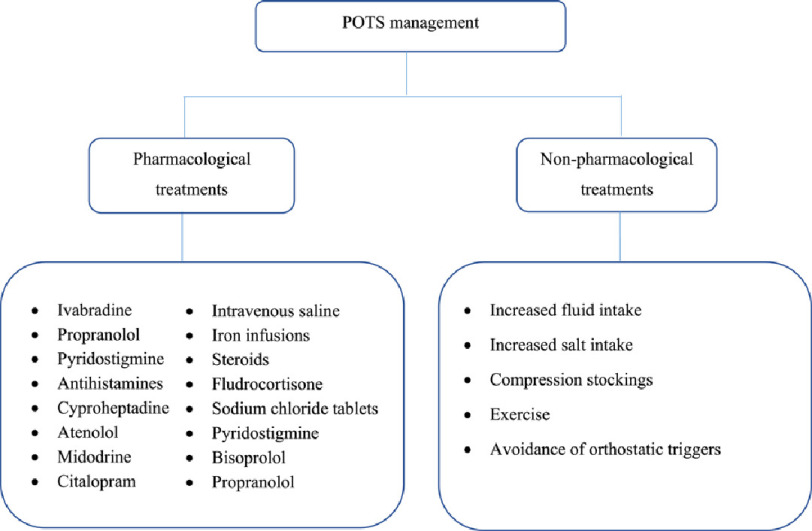
A summary of POTS treatments used in COVID-19 Pharmacological treatments.

Based on the studies included in this review, the treatment of POTS in long COVID-19 patients was based on both non-pharmacologic and pharmacologic treatments as summarized in Figure 1. Non-pharmacological approaches included exercise, fluid and salt intake, compression stockings, and avoidance of orthostatic triggers. Pharmacological treatment included ivabradine, fludrocortisone, midodrine, and antihistamines.

## Discussion

COVID-19 is a multi-organ disease with a broad spectrum of manifestations, including neurological manifestations, indicating the potential of COVID-19 to invade the nervous system^37,38^. In addition to the acute phase of SARS-COV-2 infection, reports have emerged on COVID-19 long term effects and complications^4^. More than 50% of survivors have ongoing symptoms several months after the acute infectious period^5^, and many of these symptoms are autonomic in nature^3^. The post-acute COVID-19 condition is termed post-acute COVID-19 syndrome or long COVID-19. This phenomenon is characterized by the persistence of symptoms and/or delayed complications beyond 3 or 4 weeks from the onset of the acute phase of COVID-19^39,40^. Based on recent literature, the presence of symptoms from 4-12 weeks beyond the acute infectious period of COVID-19 is termed subacute or ongoing symptoms, and post-acute COVID-19 syndrome includes symptoms persisting for *>* 12 weeks.

Long COVID-19 includes both ongoing symptoms and post-acute COVID-19 syndrome^3,37,41^. The most-commonly reported symptoms are fatigue, headache, cognitive impairment, dyspnea, palpitations, and orthostatic intolerance^3^. According to a systematic review and meta-analysis reporting the long-term effects of COVID-19, five symptoms were noted as the most common among COVID-19 survivors; fatigue (58%), headache (44%), attention disorder (27%), hair loss (25%), and dyspnea (24%)^42^. Long COVID symptoms have been reported in COVID-19 survivors admitted to hospital with severe outcomes, as well as in patients with mild disease^4,5,43,44^.

In a study of 143 severe COVID-19 survivors in Italy, 53% were female and 87% reported symptoms at 60 days. Fatigue was present in 53% of patients, breathing difficulty in 43%, and chest pain in 22%^45^. A survey of 274 non-hospitalized COVID-19 survivors in the US reported that 1/3 (52% female) had not returned to their usual state of health 2–3 weeks after the stage of acute infection. The most frequently reported symptoms include fatigue, present in 71%, cough in 61%, and headache in 61% of survivors^43^. Similarly, another study of 150 survivors of non-critical COVID-19 in France showed persistent symptoms in two-thirds of patients at 60 days of follow-up^46^. Other studies have reported the persistence of autonomic symptoms for over 100 days after the onset of acute infection. Autonomic symptoms include tachycardia upon mild exercise or standing, and temperature dysregulation^3^.

Risk factors for long COVID-19 complications are not fully understood. However, recent studies suggested that female sex and increasing age are risk factors for long COVID-19^47^. The presence of more than five symptoms in the acute phase of infection and the presence of comorbidities are also suggested to increase the risk of developing long COVID-19^48^.

The aetiology of long COVID remains unknown. However, some factors are suspected to contribute to the persistence of symptoms, including immune response or autoantibody generation, varying extent of injury, and varying time required in each organ system recovery^48,49^. Deconditioning and psychological issues may also result in the underlying symptoms^49,50^.

Autonomic dysfunction was suggested to be a possible post-acute neurological complication, explaining some of the persistent symptoms observed in long COVID^6,51,52^. This review focuses on POTS as the most frequent dysautonomia that has been reported in post COVID-19 patients.

After reviewing the literature, we found that the age of post COVID-19 POTS ranged from 22 to 59 years, and female patients outnumbered male patients. Prior studies have noted that the onset of POTS typically occurs in 12- to 50-year-old females with a ratio ranging from 4:1 to 5:1^53,54^. A lower ratio was reported in pediatric population (3.45:1)^54^.

The most common symptoms of POTS include fatigue, lightheadedness, palpitations, chest pain, orthostatic intolerance, exercise intolerance, and cognitive impairment (brain fog). These symptoms have been reported in previous studies as the most common symptoms in the initial presentation of POTS^16,55,56^.

The pathophysiology of POTS remains unknown, however there is a number of physiological mechanisms that have been supposed to be involved. These may include sympathetic dysregulation, hypovolemia, hyperadrenergic stimulation, deconditioning^12,57^, autoantibody mediated response^57^, and mast cell activation^58,59^. Moreover, COVID-19 associated manifestations may be a factor contributing to deconditioning and hypovolemia^5,60^.

The heterogeneity of POTS symptoms may complicate the diagnosis and divert it towards other disorders with similar manifestations, such as orthostatic hypotension, hyperthyroidism and anxiety^61^. In this review, the autonomic function of patients was mostly evaluated using the head up tilt test (HUT) and the active stand test. Valsalva test (with HUT) and quantitative sudomotor axon reflex tests (QSART) were also performed. HUT is considered as a golden standard for POTS diagnosis^61^. Additional tests may also be performed. These tests include the active standing test, used for initial screening and when there is a lacking access to autonomic laboratory equipment^61^. Other tests used as a confirmatory tests include 24 h ECG monitoring, used to discriminate POTS diagnosis from inappropriate sinus tachycardia^62^, and Valsalva manoeuvre, suggested to discriminate hyperadrenergic type from other forms of POTS^61,63,64^.

Moreover, variability in POTS-related symptoms, poorly explored aetiologies, and variable response to treatment make POTS management challenging and are responsible for the limited data on effective therapies. Generally, the management of POTS is used to alleviate symptoms^54,64^.

POTS management includes nonpharmacological and pharmacological interventions. Non-pharmacological treatments are recommended for all patients with POTS^22^, and have been demonstrated to be sufficiently effective in some cases^65^. Exercise, increased fluid and salt intake, and avoidance of orthostatic triggers are the most frequently reported non-pharmacological interventions in post COVID-19 POTS cases. Single or combination pharmacologic therapies were employed in the reported post COVID-19 cases. These include fludrocortisone, midodrine, antihistamines and ivabradine. Generally, pharmacologic therapies are directed at increasing intravascular volume, increasing peripheral vasoconstriction and modulating HR^54^. However, there still no robust evidence on the effectiveness of many drugs^30^.

## Conclusion

Long term effects of COVID-19 are increasingly described in the literature and present a compromising risk for quality of life and health care systems. This review identified cases with long COVID manifestations and diagnosed to have POTS, and reported clinical characteristics, diagnosis modalities and involved therapies. Data obtained in this review can be used in optimizing and promoting surveillance of POTS disorder in populations with a history of confirmed, or of a highly suspected COVID-19.
